# Cognitive Symptoms Across Diverse Cancers

**DOI:** 10.1001/jamanetworkopen.2024.30833

**Published:** 2024-08-28

**Authors:** Samantha J. Mayo, Kim Edelstein, Eshetu G. Atenafu, Rand Ajaj, Madeline Li, Lori J. Bernstein

**Affiliations:** 1 Lawrence Bloomberg Faculty of Nursing, University of Toronto, Toronto, Ontario, Canada; 2 Department of Supportive Care, Princess Margaret Cancer Centre, Toronto, Ontario, Canada; 3 Department of Psychiatry, University of Toronto, Toronto, Ontario, Canada; 4 Department of Biostatistics, Princess Margaret Cancer Centre, Toronto, Ontario, Canada

## Abstract

**Importance:**

Psychosocial health services for adults with cancer should include support for cognitive symptoms and symptom clusters.

**Objective:**

To characterize the frequency and severity of cognitive symptoms and to identify demographic and clinical risk factors associated with moderate to severe cognitive symptoms among outpatient adults with cancer seeking psychosocial support.

**Design, Setting, and Participants:**

This cross-sectional study analyzed data from routine patient-reported symptom screening during clinic appointments at the Princess Margaret Cancer Centre in Toronto, Canada, between January 1, 2013, and December 31, 2019. Participants were outpatient adults (aged ≥18 years) with diverse cancer diagnoses who endorsed interest in receiving psychosocial support from a health care team member. Data analysis was performed from April 2020 to June 2024.

**Main Outcomes and Measures:**

The presence and severity of cognitive symptoms as self-rated by participants were characterized across 12 cancer types: brain or central nervous system (CNS), breast, gastrointestinal, head and neck, gynecological, thyroid, lung and bronchus, sarcoma, genitourinary, melanoma, hematological, and all other cancers. Multivariable logistic regression was used to explore the associations between demographic, clinical, and symptom factors and moderate to severe cognitive symptoms.

**Results:**

Across the sample of 5078 respondents (2820 females [55.5%]; mean [SD] age at time of survey, 56.0 [14.1] years) requesting psychosocial support, 3480 (68.5%) reported cognitive symptoms of any severity, ranging from 59.5% in sarcoma to 86.5% in brain or CNS cancer. Moderate to severe cognitive symptoms were reported by 1544 patients (30.4%), with the proportions being 51.3% for patients with brain or CNS, 37.0% for breast, 36.2% for thyroid, 30.9% for melanoma, 29.6% for head and neck, 28.3% for gastrointestinal, 28.2% for hematological, 28.1% for gynecological, 24.9% for lung and bronchus, 24.9% for sarcoma, 21.0% for genitourinary, and 26.8% for all other cancers. Across the entire sample, moderate to severe cognitive symptoms were associated with recurrence or progression involving the CNS (odds ratio [OR], 2.62; 95% CI, 1.80-3.81), depression (OR, 1.92; 95% CI, 1.59-2.31), tiredness (OR, 1.82; 95% CI, 1.52-2.19), drowsiness (OR, 1.64; 95% CI, 1.39-1.93), anxiety (OR, 1.57; 95% CI, 1.30-1.89), shortness of breath (OR, 1.38; 95% CI, 1.16-1.61), female sex (OR, 1.33; 95% CI, 1.14-1.56), first-line chemotherapy received (OR, 1.22; 95% CI, 1.05-1.41), and metastatic disease at diagnosis (OR, 0.74; 95% CI, 0.61-0.89). Within individual cancer types, tiredness and depression were consistently associated with moderate to severe cognitive symptoms.

**Conclusions and Relevance:**

This cross-sectional study found that cognitive symptoms were frequently reported by patients across a wide range of cancer types; higher severity of cognitive symptoms was consistently associated with higher symptom burden. The findings could be used to inform decision-making regarding access to cognitive screening, assessment, and supportive care in outpatient oncology clinics.

## Key Points

**Question:**

How common are cognitive symptoms in adults with diverse cancer types?

**Findings:**

In this cross-sectional study of 5078 survey respondents with cancer seeking psychosocial support, over half of these patients reported having cognitive symptoms of any severity. A third of these patients reported moderate to severe cognitive symptoms, which were associated with disease, treatment, and other symptoms.

**Meaning:**

Findings of this study suggest that higher severity of cognitive symptoms was consistently associated with higher symptom burden; these findings could be used to inform decision-making regarding access to cognitive screening, assessment, and supportive care in outpatient oncology clinics.

## Teaser

This cross-sectional study describes the prevalence and severity of cognitive symptoms self-reported by outpatient adults with different cancer diagnoses.

## Introduction

Cognitive symptoms are distressing for many individuals with cancer, affecting emotional, social, and functional well-being.^1,2^ Common cognitive symptoms in this population include memory, attention, concentration, processing speed, and executive functioning difficulties. Although the relationship between self-reported cognitive functioning and neurocognitive test performance can vary, patients experiencing difficulties describe problems with adherence to self-care regimens, engagement in desired activities, and everyday tasks. Patient-reported difficulties in treatment and follow-up clinics can be the impetus for specialized assessment and supportive care interventions. Cognitive symptoms affect up to 75% of patients during active treatment and persist for some survivors for months to years afterward.^3,4^ However, supportive care for cognitive symptoms remains an unmet need among patients with diverse cancer diagnoses.^5,6^

One explanation for this practice gap is the relatively narrow scope of evidence to date, which has focused predominantly on patients with central nervous system (CNS) and breast cancers.^3,7,8^ Patients with primary or metastatic CNS disease may be particularly at risk for cognitive impairment associated with tumor location and treatments.^9,10^ In breast cancer, cognitive symptoms have been associated with chemotherapy,^11,12^ with evidence that anthracyclines may pose greater risk.^13^ There has been a rise in studies focusing on other cancers,^7,14,15,16,17^ but heterogeneity in definition and measurement of cognitive dysfunction, study design, and sample size makes comparability across studies challenging.^3,4,15,16,18,19,20^ In addition, research on selected cancer types used varied measurement tools and approaches to identify demographics (eg, age and sex), clinical factors (eg, time since treatment and treatment type), and co-occurring symptoms (eg, fatigue and depression) associated with cognitive impairment.^15,18^ Whether these associations are stable across cancer types is unclear, further complicating the implementation of system-level assessment and management strategies.

In an anonymous online survey of 3108 US cancer survivors who were recruited through social media,^21^ 46% of participants experienced cognitive symptoms, with high rates of symptoms among those with CNS (81%) and breast (58%) cancers as well as non-Hodgkin lymphoma (50%), Hodgkin lymphoma (50%), colorectal cancer (46%), and head and neck cancer (44%). In the survey, cognitive symptoms were associated with having received chemotherapy and concurrent report of depressive symptoms.^21^ Another study investigated cognitive symptoms among 6786 volunteer participants in the Dutch PROFILES (Patient Reported Outcomes Following Initial Treatment and Long-term Evaluation of Survivorship) cancer registry and healthy matched controls.^22^ With the exception of survivors with melanoma and basal cell or squamous cell carcinomas, survivors reported lower cognitive functioning compared with controls, as measured by the European Organisation for Research and Treatment of Cancer Quality of Life Questionnaire (QLQ-C30) Cognitive Functioning subscale. Across the sample, younger age (<50 years) was associated with poorer cognitive functioning, and clinically important cognitive symptoms were associated with greater fatigue and depression.^22^ While existing evidence suggests the need for health services to address cognitive symptoms, the research contexts in which those data were collected differ from clinical contexts in which such services would be developed and implemented. The present study investigated cognitive symptoms data obtained through an institutional routine patient-reported outcomes–based symptom screening program in an outpatient clinical setting. Using these clinical data, we aimed to (1) characterize the frequency and severity of cognitive symptoms and (2) identify demographic and clinical risk factors associated with moderate to severe cognitive symptoms among outpatient adults with cancer seeking psychosocial support.

## Methods

### Study Design

The University Health Network and University of Toronto Research Ethics Boards approved this retrospective cross-sectional study, which was conducted according to the Declaration of Helsinki.^23^ As it was impracticable to seek informed consent from individuals in this retrospective study, University Health Network Research Ethics Board waived the informed consent requirement. We followed the Strengthening the Reporting of Observational Studies in Epidemiology (STROBE) reporting guideline.^24^

### The DART

In 2010, Princess Margaret Cancer Centre in Toronto, Ontario, Canada, established the Distress Assessment and Response Tool (DART) to facilitate routine symptom screening as part of standard clinical care.^25^ The DART is a computerized survey designed to identify physical, emotional, and practical concerns using validated patient-reported outcome measures and individual items, the combination of which are ascertained at the scheduled visit through embedded computer adaptive testing algorithms.^25^ At the time of the study, patients completed the DART on a touchscreen computer in the waiting room; reports are provided to the clinic team to facilitate further assessment and intervention. At 3-month intervals, the DART includes a screening question regarding the need for psychosocial support for social difficulties: In the past month have you had concerns in the following areas that you want to discuss with a member of our health care team: family life and social activities, eg, communication, isolation, trouble concentrating, plans to have a family, sexual matters? A yes response triggers additional questions, including an item regarding cognitive symptoms. In 2013, the DART was implemented across all ambulatory cancer clinics within Princess Margaret Cancer Centre.

### Participants

We identified patients who completed the DART between January 1, 2013, and December 31, 2019, endorsed interest in receiving psychosocial support from a health care team member for social difficulties, and responded to the cognitive symptom item. For these respondents, linked clinical data from the institutional registry were retrieved to confirm cancer diagnosis and other medical and clinical characteristics concurrent with the time of response to the cognitive symptom question. If patients responded to the cognitive symptom item more than once, the most recent response was used. We excluded patients for whom a cancer diagnosis could not be confirmed, whose registry records were unavailable, or with a history of multiple primary cancers to enable attribution of characteristics (eg, treatment) to individual cancer types.

### Variables

An adapted version of the Self and Others subscale of the Social Difficulties Inventory-21^26,27^ was modified to include the following question regarding cognitive symptoms: Have you had any difficulty with thinking abilities, such as concentration, memory or word finding? The wording of this item was designed to align with the Patient-Reported Outcomes Measurement Information System Cognitive Function item bank^28^ and the QLQ-C30 Cognitive Functioning subscale,^29^ which are widely used in cancer populations.^29,30,31^ Response categories to this item included no difficulty, a little, quite a bit, and very much. For this analysis, we defined any presence of cognitive symptoms as a response of a little, quite a bit, or very much; moderate to severe cognitive symptoms were considered present from the response of quite a bit or very much.

Physical and emotional symptoms were captured in the DART with the revised Edmonton Symptom Assessment System (ESAS).^32^ We extracted ESAS severity scores for 8 common cancer-related symptoms, including tiredness, depression, pain, nausea, anxiety, drowsiness, appetite, and shortness of breath. The ESAS symptom severity is rated on a numerical scale, from 0 indicating no symptom, 4 or higher indicating moderate symptom severity, to 10 indicating worst symptom severity.^33^

Demographic and medical characteristics were obtained from the institutional cancer registry. Extracted data included date of birth; sex; primary cancer type (with *International Classification of Diseases for Oncology, Third Edition*^34^ codes); date of primary cancer diagnosis; presence of metastatic disease at the time of diagnosis; and treatment modalities used in the first course or initial management of disease, including surgery, radiotherapy, chemotherapy, hormonal therapy (eg, tamoxifen and dexamethasone), and/or biological response modifiers (eg, trastuzumab and rituximab). Cancer recurrence was categorized as involving the CNS (brain metastases or progression of primary CNS cancer), not involving the CNS (non-CNS distant metastases or local or regional to the non-CNS primary cancer site), or none. The 12 cancer types in the sample were brain or CNS, breast, gastrointestinal, genitourinary, gynecological, head and neck, hematological, lung and bronchus, melanoma, sarcoma, thyroid, and all other cancers.

### Statistical Analysis

The presence and severity of cognitive symptoms across different cancer types, demographics, and medical characteristics were described using frequencies and proportions. To identify risk factors associated with moderate to severe cognitive symptoms, we used generalized linear mixed modeling for binary outcomes across the total sample, with cancer type as a random effect to account for within-site similarity. First, we used bivariate analyses to assess the associations between moderate to severe cognitive symptoms and the following variables: age at time of survey, sex, first-line treatments received (surgery, radiotherapy, chemotherapy, hormonal therapy, or biological response modifiers), metastatic disease at diagnosis, cancer recurrence involving the CNS, and time since primary cancer diagnosis. Second, we simultaneously entered variables associated with moderate to severe cognitive symptoms in the multivariable model and reduced them to generate the most parsimonious model.

All *P* values were 2-sided, with *P* < .05 considered statistically significant. Analyses were conducted from April 2020 to June 2024 using SAS 9.4 (SAS Institute Inc).

## Results

### Sample Characteristics

A total of 5078 individuals responded to the cognitive symptoms item in the DART and had a corresponding record in the cancer registry. Patients included 2820 females (55.5%) and 2258 males (44.5%), with a mean (SD) age at time of survey of 56.0 (14.1) years (Table 1). Among these patients, 2660 (52.4%) received chemotherapy as the first-line treatment, 915 (18.0%) had metastatic disease at diagnosis, and the mean (SD) time since primary cancer diagnosis was 4.3 (4.1) years. The sample included 12 cancer types: 824 breast (16.2%), 763 hematological (15.0%), 583 gynecological (11.5%), 585 head and neck (11.5%), 499 gastrointestinal (9.8%), 348 genitourinary (6.9%), 334 lung and bronchus (6.6%), 273 brain or CNS (5.4%), 205 sarcoma (4.0%), 185 thyroid (3.6%), 94 melanoma (1.9%), and 385 other cancers (7.6%). Cancer recurrence was documented in 708 patients (13.9%), of which 318 (32.7%) involved the CNS, with 45 patients developing brain metastases and 273 having primary brain tumor progression. Further details are provided in Table 1 and eTables 1 to 3 in Supplement 1.

**Table 1.** Sample Characteristics

Parameter	Respondents, No. (%) (N = 5078)
Sex	
Female	2820 (55.5)
Male	2258 (44.5)
Age at time of survey, y	
Mean (SD)	56.0 (14.1)
Median (range)	57.1 (18.3-93.2)
<40	773 (15.2)
40-64	2808 (55.3)
≥65	1497 (29.5)
Cancer type	
Breast	824 (16.2)
Hematological	763 (15.0)
Head and neck	585 (11.5)
Gynecological	583 (11.5)
Gastrointestinal	499 (9.8)
Genitourinary	348 (6.9)
Lung and bronchus	334 (6.6)
Brain or CNS	273 (5.4)
Sarcoma	205 (4.0)
Thyroid	185 (3.6)
Melanoma	94 (1.9)
All other cancers^a^	385 (7.6)
Time since primary cancer diagnosis, y	
Mean (SD)	4.3 (4.1)
Median (range)	2.9 (0-47.0)
Metastatic disease at diagnosis	915 (18.0)
First-line treatments received	
Surgery	3060 (60.3)
Radiotherapy^b^	2750 (54.2)
Chemotherapy	2660 (52.4)
Hormonal therapy^c^	1004 (19.8)
BRM^d^	623 (12.3)
Cancer recurrence	
CNS involvement: metastases or progression	318 (32.7)
Non-CNS involvement: distant–other	377 (38.8)
Non-CNS involvement: local	121 (12.4)
Non-CNS involvement: regional	155 (15.9)
Non-CNS involvement: unknown	2 (0.2)
Time since recurrence, mean (SD), y	2.80 (4.12)
ESAS symptom severity score, mean (SD)	
Tiredness	4.12 (2.85)
Depression	3.20 (2.93)
Pain	2.49 (2.67)
Nausea	0.99 (1.96)
Anxiety	3.55 (2.99)
Drowsiness	2.87 (2.80)
Appetite	1.88 (2.64)
Shortness of breath	1.86 (2.45)

Abbreviations: BRM, biological response modifiers; CNS, central nervous system; ESAS, Edmonton Symptom Assessment System (scale: 0 [no symptom], ≥4 [moderate symptom severity], 10 [worst symptom severity]).

^a^ All other cancers include trachea; thymus; heart, mediastinum, or pleura; other and ill-defined types of the respiratory system and intrathoracic organs; selected skin, peripheral, and autonomic nervous system; retroperitoneum and peritoneum; and placenta.

^b^ Includes beam radiation, radioactive implants, and radioisotopes.

^c^ Examples: dexamethasone, prednisone, tamoxifen, letrozole, bicalutamide, and leuprolide acetate.

^d^ Examples: transtuzumab, rituximab, thalidomide, asparaginase, ibrutinib, interferon, and sorafenib.

### Cognitive Symptoms by Cancer Type

Cognitive symptoms were reported by 3480 of 5078 respondents (68.5%). The highest proportions of these patients had brain or CNS (86.5%) and breast (78.4%) cancers, with a range of 59.5% to 69.5% of patients for the other cancer types (Table 2).

**Table 2.** Frequency and Severity of Cognitive Difficulty by Cancer Type^a^

Cancer type	Respondents, No. (%) (N = 5078)	Frequency of cognitive symptoms, No. (%)^b^	Frequency of moderate to severe cognitive symptoms, No. (%)^c^	Severity of cognitive symptoms, No. (%)			
				No difficulty	A little	Quite a bit	Very much
Brain or CNS	273 (5.4)	236 (86.5)	140 (51.3)	37 (13.6)	96 (35.2)	65 (23.8)	75 (27.5)
Breast	824 (16.2)	646 (78.4)	305 (37.0)	178 (21.6)	341 (41.4)	204 (24.8)	101 (12.3)
Melanoma	94 (1.9)	65 (69.5)	29 (30.9)	29 (30.9)	36 (38.3)	16 (17.0)	13 (13.8)
Gastrointestinal	499 (9.8)	346 (69.3)	141 (28.3)	153 (30.7)	205 (41.1)	95 (19.0)	46 (9.2)
Head and neck	585 (11.5)	389 (66.5)	173 (29.6)	196 (33.5)	216 (36.9)	115 (19.7)	58 (9.9)
Gynecological	583 (11.5)	385 (66.0)	164 (28.1)	198 (34.0)	221 (37.9)	110 (18.9)	54 (9.3)
Lung and bronchus	334 (6.6)	218 (65.3)	83 (24.9)	116 (34.7)	135 (40.4)	56 (16.8)	27 (8.1)
Thyroid	185 (3.6)	120 (64.9)	67 (36.2)	65 (35.1)	53 (28.7)	37 (20.0)	30 (16.2)
Hematological	763 (15.0)	495 (64.9)	215 (28.2)	268 (35.1)	280 (36.7)	145 (19.0)	70 (9.2)
Genitourinary	348 (6.9)	210 (60.3)	73 (21.0)	138 (39.7)	137 (39.4)	50 (14.4)	23 (6.6)
Sarcoma	205 (4.0)	122 (59.5)	51 (24.9)	83 (40.5)	71 (34.6)	33 (16.1)	18 (8.8)
All other cancers^d^	385 (7.6)	248 (64.4)	103 (26.8)	137 (35.6)	145 (37.9)	66 (17.1)	37 (9.6)

Abbreviation: CNS, central nervous system.

^a^ Based on responses to the question of “During the past month, have you had any difficulty with thinking abilities, such as concentration, memory, and word finding?”

^b^ From response of a little, quite a bit, or very much.

^c^ From response of quite a bit or very much.

^d^ All other cancers include trachea; thymus; heart, mediastinum, or pleura; other and ill-defined types of the respiratory system and intrathoracic organs; selected skin, peripheral, and autonomic nervous system; retroperitoneum and peritoneum; and placenta.

A total of 1544 respondents (30.4%) reported moderate to severe cognitive symptoms, with the highest proportions of patients with brain or CNS (51.3%), breast (37.0%), and thyroid (36.2%) cancers. Proportions for the other cancer types were as follows: 30.9% for melanoma, 29.6% for head and neck, 28.3% for gastrointestinal, 28.2% for hematological, 28.1% for gynecological, 24.9% for lung and bronchus, 24.9% for sarcoma, 21.0% for genitourinary, and 26.8% for all other cancers (Table 2, Figure).

**Figure.**
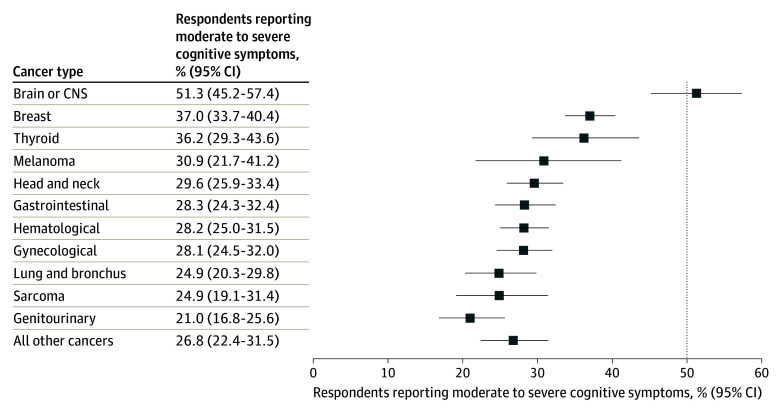
Proportion of Respondents Reporting Moderate to Severe Cognitive Difficulty by Cancer Type (N = 5078) Error bars represent 95% CIs. CNS indicates central nervous system.

### Factors Associated With Moderate to Severe Cognitive Symptoms Across All Cancer Types

On bivariate analysis, moderate to severe cognitive symptoms were associated with all ESAS symptoms assessed. Associations with depression (odds ratio [OR], 4.35; 95% CI, 3.82-4.95) and tiredness (OR, 4.29; 95% CI, 3.73-4.92) were of the greatest magnitude (Table 3). Moderate to severe cognitive symptoms were also associated with recurrence involving the CNS (OR, 2.60; 95% CI, 1.82-3.71), female sex (OR, 1.49; 95% CI, 1.29-1.73), 40 to 64 years of age (OR, 1.26; 95% CI, 1.05-1.51), and chemotherapy as the first-line treatment received (OR, 1.17; 95% CI, 1.03-1.34). Survivors with more than 10 years since diagnosis tended to have lower odds of moderate to severe cognitive symptoms compared with those with less than 2 years since diagnosis (OR, 0.71; 95% CI, 0.54-0.94). eTable 3 in Supplement 1 provides additional details.

**Table 3.** Bivariate Analysis of Moderate to Severe Cognitive Symptoms

Factor	Respondents, No. (%)^a^	Moderate to severe cognitive symptoms^b^	
		OR (95% CI)	*P* value
First-line treatments received			
Surgery			
No	2018 (39.7)	1 [Reference]	.71
Yes	3060 (60.3)	1.03 (0.88-1.21)	
Radiotherapy			
No	2328 (45.8)	1 [Reference]	.54
Yes	2750 (54.2)	1.04 (0.91-1.19)	
Chemotherapy			
No	2418 (47.6)	1 [Reference]	.02
Yes	2660 (52.4)	1.17 (1.03-1.34)	
Hormone therapy			
No	4074 (80.2)	1 [Reference]	.78
Yes	1004 (19.8)	1.03 (0.85-1.24)	
BRM			
No	4455 (87.7)	1 [Reference]	.60
Yes	623 (12.3)	1.06 (0.86-1.29)	
Cancer recurrence			
None	655 (12.9)	1 [Reference]	<.001
Non-CNS involvement^c^	4105 (80.8)	0.89 (0.74-1.08)	
CNS involvement	318 (6.3)	2.60 (1.82-3.71)	
Metastatic disease at diagnosis^d^			
No	4163 (82.0)	1 [Reference]	.06
Yes	915 (18.0)	0.85 (0.71-1.01)	
Sex			
Female	2820 (55.5)	1.49 (1.29-1.73)	<.001
Male	2258 (44.5)	1 [Reference]	
Age at time of survey, y			
<40	773 (15.2)	1 [Reference]	.02
40-64	2808 (55.3)	1.26 (1.05-1.51)	
≥65	1497 (29.5)	1.10 (0.90-1.34)	
Time since primary cancer diagnosis, y			
<2	1369 (27.0)	1 [Reference]	.07
2-5	2711 (53.4)	0.89 (0.77-1.02)	
6-10	650 (12.8)	0.94 (0.77-1.16)	
>10	345 (6.8)	0.71 (0.54-0.94)	
Missing data	3 (0.1)		
ESAS tiredness score			
<4	2318 (45.9)	1 [Reference]	<.001
≥4	2734 (54.1)	4.29 (3.73-4.92)	
Missing data	26 (0.5)		
ESAS depression score			
<4	2970 (58.7)	1 [Reference]	<.001
≥4	2090 (41.3)	4.35 (3.82-4.95)	
Missing data	18 (0.4)		
ESAS pain score			
<4	3541 (70.0)	1 [Reference]	<.001
≥4	1518 (30.0)	2.65 (2.33-3.01)	
Missing data	19 (0.4)		
ESAS anxiety score			
<4	2764 (54.7)	1 [Reference]	<.001
≥4	2293 (45.3)	3.97 (3.49-4.52)	
Missing data	21 (0.4)		
ESAS nausea score			
<4	4505 (89.3)	1 [Reference]	<.001
≥4	540 (10.7)	2.38 (1.98-2.86)	
Missing data	33 (0.6)		
ESAS appetite score			
<4	3881 (76.9)	1 [Reference]	<.001
≥4	1169 (23.2)	2.66 (2.31-3.06)	
Missing data	28 (0.6)		
ESAS drowsiness score			
<4	3215 (63.7)	1 [Reference]	<.001
≥4	1835 (36.3)	3.70 (3.26-4.21)	
Missing data	28 (0.6)		
ESAS shortness of breath score			
<4	3925 (77.7)	1 [Reference]	<.001
≥4	1125 (22.3)	2.91 (2.53-3.36)	
Missing data	28 (0.6)		

Abbreviations: BRM, biological response modifiers; CNS, central nervous system; ESAS, Edmonton Symptom Assessment System (scale: 0 [no symptom], ≥4 [moderate symptom severity], 10 [worst symptom severity]); OR, odds ratio.

^a^ Percentages for missing data were calculated from complete data (N = 5078) to show the proportion of missing, whereas all other percentages were calculated excluding the missing data to add up to 100%.

^b^ High cognitive difficulty (from response of quite a bit or very much).

^c^ Includes distant–other, local, regional, and unknown.

^d^ Patients with primary brain cancers were not considered to have metastatic disease.

Multivariable analysis showed that moderate to severe cognitive symptoms were associated with recurrence involving the CNS (OR, 2.62; 95% CI, 1.80-3.81), depression (OR, 1.92; 95% CI, 1.59-2.31), tiredness (OR, 1.82; 95% CI, 1.52-2.19), drowsiness (OR, 1.64; 95% CI, 1.39-1.93), anxiety (OR, 1.57; 95% CI, 1.30-1.89), shortness of breath (OR, 1.38; 95% CI, 1.16-1.61), female sex (OR, 1.33; 95% CI, 1.14-1.56), first-line chemotherapy (OR, 1.22; 95% CI, 1.05-1.41), and metastatic disease at diagnosis (OR, 0.74; 95% CI, 0.61-0.89) (Table 4). Moderate to severe cognitive symptoms were not associated with age at time of survey or time since diagnosis. eTable 4 in Supplement 1 provides additional details.

**Table 4.** Multivariable Analysis of Moderate to Severe Cognitive Symptoms

Factor	Moderate to severe cognitive symptoms^a^	
	OR (95% CI)	*P* value
Sex		
Male	1 [Reference]	<.001
Female	1.33 (1.14-1.56)	
Age at time of survey, y		
<40	1 [Reference]	.31
40-64	1.17 (0.96-1.42)	
≥65	1.01 (0.89-1.39)	
Time since primary cancer diagnosis, y		
<2	1 [Reference]	.18
2-5	1.01 (0.86-1.18)	
6-10	1.05 (0.84-1.32)	
>10	0.74 (0.55-1.00)	
Metastatic disease at diagnosis: yes	0.74 (0.61-0.89)	.002
First-line chemotherapy received	1.22 (1.05-1.41)	.009
Cancer recurrence		
None	1 [Reference]	<.001
Non-CNS involvement^b^	0.81 (0.66-1.00)	
CNS involvement	2.62 (1.80-3.81)	
ESAS symptom severity score ≥4		
Depression	1.92 (1.59-2.31)	<.001
Tiredness	1.82 (1.52-2.19)	<.001
Drowsiness	1.64 (1.39-1.93)	<.001
Anxiety	1.57 (1.30-1.89)	<.001
Shortness of breath	1.38 (1.16-1.61)	<.001

Abbreviations: CNS, central nervous system; ESAS, Edmonton Symptom Assessment System (scale: 0 [no symptom], ≥4 [moderate symptom severity], 10 [worst symptom severity]).

^a^ Moderate to severe cognitive symptoms (from response of quite a bit or very much).

^b^ Includes distant–other, local, regional, and unknown.

### Factors Associated With Moderate to Severe Cognitive Symptoms in Individual Cancer Type

The consistency of cognitive risk factors across individual cancer types was explored through post hoc stratified analyses. In each of the 12 cancer types, the multivariable model was replicated and a reduced parsimonious model was generated based on statistically significant factors. Sex was omitted from the model for breast and gynecological cancer groups, and CNS-involved recurrence was omitted for brain or CNS cancer, as all cases had documented recurrence involving the CNS. In all disease types, moderate to severe cognitive symptoms were consistently associated with ESAS symptom severity, especially depression (ORs ranging from 1.81 [95% CI, 1.11-2.97] for hematological cancer to 4.04 [95% CI, 2.57-6.36] for gynecological cancer) and tiredness (ORs ranging from 1.66 [95% CI, 1.17-2.36] for breast cancer to 5.36 [95% CI, 1.67-17.28] for thyroid cancer). Moderate to severe cognitive symptoms were also associated with shortness of breath severity in 3 of the cancer groups: head and neck (1.81; 95% CI, 1.13-2.90), breast (2.54; 95% CI, 1.67-3.85), and all other cancers (2.06; 95% CI, 1.18-3.62) (eTable 5 in Supplement 1).

In some cancer types, some cancer and demographic factors remained associated with cognitive symptom severity even after controlling for ESAS symptom severity. These factors included first-line chemotherapy in breast and hematological cancers, recurrence in gynecological cancers, older age in brain or CNS cancers, and female sex in hematological and other cancers (eTable 5 in Supplement 1).

## Discussion

In routinely collected patient-reported outcomes data, 5078 patients with a wide range of primary cancers requested psychosocial support for social difficulties. Over half of these patients (68.5%) reported mild or worse cognitive symptoms, and almost one-third (30.4%) reported moderate to severe cognitive symptoms. These findings were similar within each of the 12 cancer types, with at least 60% of participants reporting cognitive symptoms and at least 20% reporting moderate to severe cognitive symptoms. By characterizing cognitive symptoms in a large clinical dataset assessed similarly across a breadth of diverse cancer types, the findings extend previous work using volunteer participants or focusing on single cancer types. Results of this study highlight the pervasiveness of cognitive symptoms in patients with cancer seeking psychosocial support, the associations with other patient-reported outcome measures, and the need to further characterize cognitive outcomes and risk factors in understudied adult cancer populations.^35^ These findings may inform the development of health services from an organizational perspective and offer hypothesis-generating data for future studies conducted within specific populations.

Cognitive symptoms were most severe in patients with CNS cancers, and moderate to severe cognitive symptoms were associated with primary CNS cancer progression or metastasis. Problems with memory, executive functions, and communication have been reported among patients with brain cancers^36,37,38,39^ and have been attributed to tumor size and location, number of lesions, rapid progression, brain edema, and the acute and late adverse effects of treatment, including radiotherapy.^38,39,40,41,42,43^ However, the presence and severity of symptoms among these patients vary.^44^ In this study, we underscored the degree to which patients with brain cancers seeking psychosocial support required support for cognitive issues, and how this may compare across patients with other cancer types. The relatively high prevalence of cognitive symptoms across patients with non-CNS cancers seeking psychosocial support and the similarity of associated factors warrant future investigation. Metastatic disease at the time of diagnosis was associated with lower odds of moderate to severe cognitive difficulties among people seeking psychosocial support. Individuals with metastatic cancer experience many moderate to severe concerns, particularly regarding worsening disease and the future,^45^ but may also express hope and reflect on favorable aspects of their lives within the context of terminal illness.^46^ Such experiences may contribute to the concerns reported when seeking psychosocial support.

Severity of concurrent ESAS symptoms were associated with reports of moderate to severe cognitive symptoms. Tiredness and depression, and to a lesser degree drowsiness and anxiety, were all independently associated with increased odds of moderate to severe cognitive symptoms in the total sample and in each of the 12 cancer types. Associations of cognitive symptoms with fatigue and emotional distress have been reported in several cancer populations,^21,22,47,48,49,50^ with speculations that their co-occurrence may constitute a symptom cluster with shared biological mechanisms, such as inflammation.^51,52^ We also found an association with shortness of breath in the overall sample and in breast and head and neck cancers. We were unable to identify whether the associated factors were parallel outcomes from cancer and treatment; causative, with cognitive decline playing a role in other factors or in the opposite direction, or some combination; or a self-reported halo,^53^ a tendency to generally rate any domain throughout all self-reported measures as more unfavorable or favorable.^51,52^ However, taken together, these findings suggest that in addition to psychoeducation and self-management strategies focusing on cognitive symptoms, interventions promoting management of concurrent symptoms, such as fatigue and emotional distress, may be beneficial.^8,54^

Receiving chemotherapy as a first-line treatment was associated with moderate to severe cognitive symptoms. The association between chemotherapy and cognitive symptoms in cancer has been consistently demonstrated,^12,15,55^ with hypothesized mechanisms underlying oxidative stress and inflammation, among others.^56,57^ However, cognitive impairment and cognitive symptoms can be observed prior to chemotherapy,^50,58^and there is evidence of cognitive changes related to other treatments and novel therapies.^59,60,61^ In a systematic review of longitudinal studies in breast cancer, cognitive decline after treatment was generally greater in patients undergoing chemotherapy compared with those not receiving chemotherapy, but cognitive decline was not exclusive to that group.^12,62^ Different treatments, such as radiotherapy, hormonal therapy, immunotherapy, and/or other targeted therapy, may affect cognition in subgroups of patients that we may not have captured, such as patients with head and neck cancer who receive incidental brain radiation and are at greater risk for cognitive impairments.^14,35^ Adding cognitive outcomes to emerging clinical trials can accelerate understanding of the potential role of novel treatment regimens, as has been recommended.^19^

The risk of moderate to severe cognitive symptoms increased among female patients in this sample, which would not necessarily be expected given that there are no sex-based differences in subjective cognitive decline in healthy adults at or over age 45 years.^63^ This finding may be partly explained by the elevated rates of moderate to severe cognitive symptoms in breast cancer, but female sex was also a significant factor in moderate to severe cognitive symptoms in patients with hematological cancers. This finding may be attributed to treatment-induced menopause or sex differences in treatment toxic effects.^64^ We lacked data on gender identity, which is relevant since symptom reporting is also affected by social factors associated with gender identity.^65^ Distinguishing the complex sex- and gender identity–based differences in cancer-related symptoms remains an understudied area of research.

In the multivariable analysis, age was not a significant risk factor for cognitive symptoms. However, because the experience and needs related to symptom management may vary across the life span, further research to characterize the patterns and outcomes of cognitive symptoms by developmental stage can inform effective age-appropriate interventions.

### Limitations

Limitations of this study include its retrospective cross-sectional design and lack of prospective data regarding other factors potentially associated with cognitive symptoms. Additionally, details on the treatments received after the primary treatment regimen were unavailable. This analysis was limited to patients who endorsed interest in receiving psychosocial support for social difficulties during routine symptom screening. Therefore, we did not have information on cognitive symptoms for those who declined psychosocial support. The prescreening questions used may account for the slightly higher prevalence of cognitive symptoms reported in this study compared with other published studies.^21^ Nonetheless, we have no reason to speculate that this method would affect the relative prevalence of cognitive symptoms across the diverse cancer types in the sample. While the findings suggest that cognitive issues are common among patients seeking psychosocial support, it is unknown whether the findings generalize to patients who do not complete the DART in clinic or do not endorse social difficulties requiring psychosocial support.

## Conclusions

In this cross-sectional study of outpatient adults with cancer requesting psychosocial support on routine symptom screening, cognitive symptoms were frequently reported. Higher severity of cognitive symptoms was consistently associated with higher symptom burden. A common limitation of the prior literature was the applicability in clinical settings vs in research trials. Our findings advance extant literature that was developed from research context by reinforcing it with a clinical dataset, supporting clinical applicability. When cognitive symptoms are not acknowledged and/or addressed by the care team, patients experience distress, fear, and stigma and face barriers to appropriate supportive care.^1,2,66^ Our findings provide clinicians with the prevalence of cognitive symptoms in a wide range of cancer types, which could be used to inform decision-making regarding access to cognitive screening, assessment, and supportive care in outpatient oncology clinics. In addition, the findings signal the need for expanded research to understand site-specific factors associated with cognitive symptoms. Various interventions have shown promise in promoting self-management and rehabilitation of cognitive deficits among patients with CNS and breast cancers.^67,68,69,70^ Continued evaluation of the potential role of such interventions in other cancer sites is warranted.

## Notes

Supplement 1. **eTable 1.** Sample Characteristics by Cancer Type **eTable 2.** Cancer Subtypes (n=5078) **eTable 3.** Bivariate Analysis of Moderate to Severe Cognitive Symptoms (Age, Time Since Diagnosis, and Symptoms as Continuous Variables) **eTable 4.** Initial Multivariable Model Including All Variables of Interest to Model Moderate to Severe Cognitive Symptoms **eTable 5.** Post-Hoc Reduced Multivariable Analyses of Moderate to Severe Cognitive Symptoms by Cancer Type

Supplement 2.
Data Sharing Statement
