# Supplementary material for: Cognitive Symptoms Across Diverse Cancers
Source: JAMA Netw Open. 2024 Aug 28;7(8):e2430833. doi: 10.1001/jamanetworkopen.2024.30833 (PMC11358862; doi:10.1001/jamanetworkopen.2024.30833)
Supplement: Supplement 1. — eTable 1. Sample Characteristics by Cancer Type eTable 2. Cancer Subtypes (n=5078) eTable 3. Bivariate Analysis of Moderate to Severe Cognitive Symptoms (Age, Time Since Diagnosis, and Symptoms as Continuous Variables) eTable 4. Initial Multivariable Model Including All Variables of Interest to Model Moderate to Severe Cognitive Symptoms eTable 5. Post-Hoc Reduced Multivariable Analyses of Moderate to Severe Cognitive Symptoms by Cancer Type [file jamanetwopen-e2430833-s001.pdf]

Supplemental Online Content

Mayo SJ, Edelstein K, Atenafu EG, Ajaj R, Li M, Bernstein LJ. Cognitive symptoms across diverse cancers. *JAMA Netw Open*. 2024;7(8):e2430833. doi:10.1001/jamanetworkopen.2024.30833

eTable 1. Sample Characteristics by Cancer Type

eTable 1~~2~~. Cancer Subtypes (n=5078)

eTable 3. Bivariate Analysis of Moderate to Severe Cognitive Symptoms (Age, Time Since Diagnosis, and Symptoms as Continuous Variables)

eTable 4. Initial Multivariable Model Including All Variables of Interest to Model Moderate to Severe Cognitive Symptoms

eTable 5. Post-Hoc Reduced Multivariable Analyses of Moderate to Severe Cognitive Symptoms by Cancer Type

This supplemental material has been provided by the authors to give readers additional information about their work.

© 2024 Mayo SJ et al. *JAMA Network Open*

eTable 1. Sample Characteristics by Cancer Type

Parameter	Total (n=5078)	Breast (n=824)	Heme (n=763)	Head/ Neck (n=585)	Gyne (n=583)	GI (n=499)	GU (n=348)	Lung/ Bronchus (n=334)	Brain/ CNS (n=273)	Sarcoma (n=205)	Thyroid (n=185)	Melanoma (n=94)	All Other Cancers (n=385)
**Sex, n (%)**													
**Female**	2820 (55.53)	824 (100)	325 (42.60)	147 (25.13)	583 (100)	197 (39.48)	20 (5.75)	174 (52.10)	118 (43.22)	103 (50.24)	133 (71.89)	40 (42.55)	156 (40.52)
**Male**	2258 (44.47)	0 (0)	438 (57.40)	438 (74.87)	0 (0)	302 (60.52)	328 (94.25)	160 (47.90)	155 (56.78)	102 (49.76)	52 (28.11)	54 (57.45)	229 (59.48)
**Age at time of survey (years)**													
**Mean (SD)**	55.98 (14.11)	54.64 (11.64)	54.48 (15.61)	59.77 (12.25)	54.23 (13.29)	59.18 (12.05)	54.18 (16.59)	64.97 (10.88)	50.57 (13.71)	52.14 (16.29)	47.18 (14.35)	56.99 (14.42)	58.29 (14.33)
**Median (range)**	57.1 (18.3-93.2)	54.3 (22.5-85.7)	56.7 (18.3-92.4)	60.6 (22.5 – 88.0)	54.9 (19.4-85.0)	60.3 (22.8-93.2)	56.9 (19.8-85.3)	65.7 (32.5-88.9)	51.5 (19.0-82.4)	52.2 (18.6-87.5)	46.1 (19.9-82.0)	58.0 (23.8-84.3)	60.2 (21.6-85.2)
**<40 years, n (%)**	773 (15.22)	89 (10.80)	168 (22.02)	40 (6.84)	95 (16.30)	35 (7.01)	92 (26.44)	8 (2.40)	64 (23.44)	54 (26.34)	66 (35.68)	14 (14.89)	48 (12.47)
**40-64 years. n (%)**	2808 (55.30)	567 (68.81)	366 (47.97)	337 (57.61)	354 (60.72)	296 (59.32)	141 (40.52)	143 (42.81)	167 (61.17)	103 (50.24)	94 (50.81)	45 (47.87)	195 (50.65)
**65+ years, n (%)**	1497 (29.48)	168 (20.39)	229 (30.01)	208 (35.56)	134 (22.98)	168 (33.67)	115 (33.05)	183 (54.79)	42 (15.38)	48 (23.41)	25 (13.51)	35 (37.23)	142 (36.88)
**Time since diagnosis (years)**													
**Mean (SD)**	4.28 (4.09)	3.95 (3.90)	5.51 (5.22)	4.19 (3.77)	4.26 (3.22)	3.15 (2.85)	4.56 (4.15)	2.9 (2.59)	4.5 (4.5)	4.25 (3.61)	4.04 (3.51)	4.0 (3.06)	5.11 (5.36)
**Median (range)**	2.85 (0-47)	2.6 (0.1-33)	3.5 (0.1-42.8)	3.0 (0.2-47)	3.4 (0.1-20.7)	2.3 (0-19.4)	3.3 (0.4-38.4)	2.1 (0-17.3)	2.6 (0-30.3)	3.1 (0.2-29.7)	2.6 (0.1-18.7)	2.8 (0.2-13.3)	3.1 (0-45.4)
**Metastatic disease at diagnosis[a]**	915 (18.02)	39 (4.73)	106 (13.89)	292 (49.91)	51 (8.75)	125 (25.05)	62 (17.82)	157 (47.01)	4 (1.47)	7 (3.41)	35 (18.92)	16 (17.02)	21 (5.45)
**First-line treatments received, n(%)**													
**Surgery**	3060 (60.26)	797 (96.72)	17 (2.23)	244 (41.71)	494 (84.73)	314 (62.93)	261 (75.00)	117 (35.03)	224 (82.05)	169 (82.44)	181 (97.84)	88 (93.62)	154 (40.00)
**Radiotherapy**	2750 (54.16)	676 (82.04)	272 (35.65)	505 (86.32)	193 (33.10)	169 (33.87)	84 (24.14)	199 (59.58)	186 (68.13)	155 (75.61)	107 (57.84)	16 (17.02)	188 (48.83)

© 2024 Mayo SJ et al. *JAMA Network Open*

Parameter	Total (n=5078)	Breast (n=824)	Heme (n=763)	Head/ Neck (n=585)	Gyne (n=583)	GI (n=499)	GU (n=348)	Lung/ Bronchus (n=334)	Brain/ CNS (n=273)	Sarcoma (n=205)	Thyroid (n=185)	Melanoma (n=94)	All Other Cancers (n=385)
**Chemotherapy**	2660 (52.38)	510 (61.89)	644 (84.40)	243 (41.54)	300 (51.46)	310 (62.12)	79 (22.70)	236 (70.66)	172 (63.00)	34 (16.59)	1 (0.54)	7 (7.45)	124 (32.21)
**Hormonal Therapy**[c]	1004 (19.77)	552 (66.99)	316 (41.42)	15 (2.56)	23 (3.95)	21 (4.21)	50 (14.37)	1 (0.30)	7 (2.56)	1 (0.49)	7 (3.78)	0 (0)	11 (2.86)
**Biological Response Modifiers**[b]	623 (12.27)	139 (16.87)	348 (45.61)	14 (2.39)	9 (1.54)	23 (4.61)	13 (3.74)	5 (1.50)	9 (3.30)	3 (1.46)	1 (0.54)	20 (21.28)	39 (10.13)
**Recurrence (n/%)**													
**CNS (Mets+progression)**	318 (32.68)	10 (10.87)	4 (4.00)	0 (0.00)	5 (4.46)	4 (4.60)	2 (4.00)	12 (26.67)	273 (100.00)	1 (2.70)	1 (6.25)	4 (14.29)	2 (3.77)
**Distant – other**	377 (38.75)	54 (58.70)	82 (82.00)	31 (38.75)	55 (49.11)	54 (62.07)	15 (30.00)	16 (35.56)	0 (0.00)	24 (64.86)	4 (25.00)	13 (46.43)	29 (54.72)
**Local (not CNS)**	121 (12.44)	17 (18.48)	8 (8.00)	26 (32.50)	10 (8.93)	18 (20.69)	14 (28.00)	8 (17.78)	0 (0.00)	5 (13.51)	2 (12.50)	2 (7.14)	11 (20.75)
**Regional**	155 (15.93)	11 (11.96)	4 (4.00)	23 (28.75)	42 (37.50)	11 (12.64)	19 (38.00)	9 (20.00)	0 (0.00)	7 (18.92)	9 (56.25)	9 (32.14)	11 (20.75)
**Unknown**	2 (0.21)	0 (0.00)	2 (2.00)	0 (0.00)	0 (0.00)	0 (0.00)	0 (0.00)	0 (0.00)	0 (0.00)	0 (0.00)	0 (0.00)	0 (0.00)	0 (0.00)
**Time since recurrence (years) n, Mean (SD)**	708, 2.80 (4.12)	92, 2.60 (2.71)	99, 4.30 (5.98)	80, 2.37 (4.96)	112, 2.61 (2.87)	87, 1.74 (1.95)	50, 4.48 (5.94)	45, 1.81 (1.83)	9, 1.65 (3.03)	37, 1.73 (2.04)	16, 3.86 (4.70)	28, 2.41 (2.35)	53, 3.23 (5.28)
**ESAS Tiredness n, Mean (SD)**	5052, 4.12 (2.85)	819, 4.02 (2.70)	755, 4.00 (2.88)	585, 4.00 (2.94)	580, 4.07 (2.85)	499, 4.44 (2.84)	344, 3.53 (2.74)	332, 4.47 (2.80)	272, 4.90 (2.93)	204, 3.94 (2.94)	185, 4.63 (2.90)	94, 3.76 (2.82)	383, 4.04 (2.78)
**ESAS Depression n, Mean (SD)**	5060, 3.20 (2.93)	822, 3.37 (2.81)	758, 3.04 (2.90)	584, 3.05 (2.94)	580, 3.30 (3.02)	499, 3.43 (3.07)	346, 2.90 (2.78)	332, 3.14 (2.84)	273, 3.53 (2.99)	202, 3.27 (3.12)	185, 3.24 (3.18)	94, 2.73 (2.76)	385, 3.08 (2.83)

© 2024 Mayo SJ et al. *JAMA Network Open*

Parameter	Total (n=5078)	Breast (n=824)	Heme (n=763)	Head/ Neck (n=585)	Gyne (n=583)	GI (n=499)	GU (n=348)	Lung/ Bronchus (n=334)	Brain/ CNS (n=273)	Sarcoma (n=205)	Thyroid (n=185)	Melanoma (n=94)	All Other Cancers (n=385)
**ESAS Pain n, Mean (SD)**	5059, 2.49 (2.67)	822, 2.54 (2.57)	758, 2.42 (2.66)	585, 2.69 (2.68)	578, 2.43 (2.65)	499, 2.78 (2.75)	346, 1.88 (2.40)	333, 2.74 (2.76)	272, 2.21 (2.82)	204, 3.23 (2.92)	185, 2.07 (2.52)	94, 2.43 (2.55)	383, 2.23 (2.58)
**ESAS Nausea n, Mean (SD)**	5045, 0.99 (1.96)	821, 0.72 (1.64)	754, 1.00 (1.93)	579, 0.91 (1.83)	579, 0.97 (1.94)	497, 1.56 (2.46)	345, 0.65 (1.56)	334, 1.25 (2.17)	271, 1.24 (2.25)	203, 1.03 (1.99)	185, 0.97 (2.04)	94, 0.65 (1.40)	383, 0.96 (1.87)
**ESAS Anxiety n, Mean (SD)**	5057, 3.55 (2.99)	822, 3.87 (2.96)	756, 3.34 (2.95)	581, 3.25 (2.96)	581, 3.88 (3.08)	499, 3.83 (3.13)	346, 3.21 (2.70)	332, 3.42 (2.87)	273, 3.79 (3.01)	204, 3.49 (3.22)	185, 3.42 (3.17)	94, 3.66 (2.79)	384, 3.22 (2.84)
**ESAS Drowsiness n, Mean (SD)**	5050, 2.87 (2.80)	821, 2.67 (2.73)	755, 2.76 (2.76)	584, 2.81 (2.78)	578, 2.75 (2.81)	499, 3.17 (2.87)	344, 2.51 (2.59)	333, 3.03 (2.77)	271, 3.76 (3.01)	204, 2.91 (2.90)	185, 3.44 (3.01)	94, 2.82 (2.91)	382, 2.72 (2.67)
**ESAS Appetite n, Mean (SD)**	5050, 1.88 (2.64)	820, 1.38 (2.21)	756, 1.77 (2.51)	583, 2.50 (3.00)	581, 1.59 (2.54)	498, 2.65 (3.02)	344, 1.18 (2.04)	334, 2.60 (2.83)	271, 2.00 (2.67)	203, 1.59 (2.44)	185, 1.66 (2.70)	94, 1.41 (2.02)	381, 1.94 (2.68)
**ESAS Shortness of Breath n, Mean (SD)**	5050, 1.86 (2.45)	820, 1.56 (2.25)	755, 1.94 (2.43)	581, 1.73 (2.38)	581, 1.45 (2.25)	498, 2.13 (2.59)	345, 1.55 (2.14)	334, 3.44 (2.82)	272, 1.72 (2.40)	203, 1.60 (2.40)	185, 1.78 (2.57)	94, 1.30 (2.01)	382, 2.04 (2.53)
**ESAS Well-being n, Mean (SD)**	5063, 3.88 (2.66)	822, 3.96 (2.51)	759, 3.71 (2.63)	585, 3.74 (2.71)	581, 3.91 (2.74)	498, 4.19 (2.77)	346, 3.41 (2.56)	333, 4.25 (2.58)	273, 4.15 (2.79)	204, 3.97 (2.66)	184, 3.72 (2.78)	94, 3.61 (2.57)	384, 3.81 (2.70)

Note:: *CNS*, Central Nervous System; *ESAS*, Edmonton Symptom Assessment System (0-10, higher score indicates worse symptoms/well-being).

[a] Metastatic disease at diagnosis: For those with referral reason as “new primary”, metastatic disease is indicated by either: i) Group stage – stage 4 (including 4, 4A, 4B, 4C), or ii) ‘pathM’/’clinicM’/’otherM’ = “1”

[b] Examples: transtuzumab (Herceptin), rituximab, thalidomide, asparaginase, ibrutinib, interferon, sorafenib

[c] Hormonal therapy includes both endocrine therapies and steroids. Examples: dexamethasone, prednisone, tamoxifen, letrozole, bicalutamide, leuprolide acetate

[d] Range 0-10, where “0- symptom is absent” to “10 – worst possible severity”

© 2024 Mayo SJ et al. *JAMA Network Open*

eTable 2. Cancer Subtypes (n=5078)

Type	Frequency	Percent
**Breast**	**824**	**16.23**
**Hematological**	**763**	**15.03**
Leukemia	296	5.83
Non-Hodgkin Lymphoma	212	4.17
Multiple Myeloma	176	3.47
Hodgkin Lymphoma	79	1.56
**Head and Neck**	**585**	**11.52**
Oral Cavity	206	4.06
Pharynx	190	3.74
Larynx	95	1.87
Salivary Glands	52	1.02
Nasal Cavity, Middle Ear and Accessory Sinuses	38	0.75
Other Head and Neck[a]	4	0.08
**Gynecological**	**583**	**11.52**
Ovary	224	4.41
Uterus	195	3.84
Cervix	112	2.21
Other Gynecological[b]	52	1.02
**Gastrointestinal**	**499**	**9.83**
Colorectal	156	3.07
Pancreas	84	1.65
Liver	76	1.50
Stomach	49	0.96
Esophagus	24	0.47
Other Gastrointestinal[c]	110	2.17
**Genitourinary**	**348**	**6.85**
Prostate	129	2.54
Testis	126	2.48
Kidney and Renal Pelvis	63	1.24
Bladder	26	0.51
Other Genitourinary[d]	4	0.08
**Lung and Bronchus**	**334**	**6.58**
**Brain/CNS**	**273**	**5.38**
**Sarcoma**	**205**	**4.04**
Soft Tissue	176	3.47
Bone	29	0.57
**Thyroid**	**185**	**3.64**
**Melanoma**	**94**	**1.85**
**All other Cancers[e]**	**385**	**7.58**

Note: CNS, Central Nervous System

[a] Examples: ill-defined types in lip, oral cavity and pharynx

[b] Examples: vulva, vagina, and unspecified female genital organs

[c] Examples: small intestine, anus, gall bladder, and ill-specified digestive organs

[d] Examples: penis, ureter, and unspecified male genital organs

[e] All other Cancers includes: trachea, thymus, heart/mediastinum/pleura, other and ill-defined types of the respiratory system and intrathoracic organs, selected skin, peripheral nerves and autonomic nervous system, retroperitoneum and peritoneum, placenta.

© 2024 Mayo SJ et al. *JAMA Network Open*

eTable 3. Bivariate Analysis of Moderate to Severe Cognitive Symptoms (age, time since diagnosis, and symptoms as continuous variables)

Effect	M (SD)	Moderate to Severe Cognitive Symptoms[a]	
		OR (95% CI)	p-value
**Age at Survey (years)**	56.0 (14.1)	1.00 (1.00 to 1.00)	0.81
**Time since Diagnosis (years)**	4.3 (4.1)	0.98 (0.97 to 1.00)	0.05
**ESAS Tiredness**	4.1 (2.8)	1.36 (1.33 to 1.40)	<0.001
**ESAS Depression**	3.2 (2.9)	1.34 (1.31 to 1.37)	<0.001
**ESAS Pain**	2.5 (2.7)	1.21 (1.19 to 1.24)	<0.001
**ESAS Nausea**	1.0 (2.0)	1.18 (1.14 to 1.22)	<0.001
**ESAS Anxiety**	3.6 (3.0)	1.32 (1.29 to 1.35)	<0.001
**ESAS Drowsiness**	2.9 (2.8)	1.30 (1.27 to 1.33)	<0.001
**ESAS Appetite**	1.9 (2.6)	1.20 (1.17 to 1.23)	<0.001
**ESAS Shortness of Breath**	1.9 (2.4)	1.22 (1.19 to 1.25)	<0.001

Note: ESAS, Edmonton Symptom Assessment System (0-10, higher score indicates worse symptoms).
[a] moderate to severe cognitive symptoms(quite a bit/very much)

© 2024 Mayo SJ et al. *JAMA Network Open*

eTable 4. Initial Multivariable Model Including all Variables of Interest to Model Moderate to Severe Cognitive Symptoms

Effect	Moderate to Severe Cognitive Symptoms[a]	
	OR (95% CI)	P
First Line Treatments Received		
Surgery (Yes)	1.12 (0.94 to 1.34)	0.19
Radiation (Yes)	1.06 (0.91 to 1.23)	0.43
Chemotherapy (Yes)	1.20 (1.02 to 1.39)	0.02
Hormone Therapy (Yes)	1.05 (0.85 to 1.30)	0.63
Biological Response Modifiers (Yes)	1.04 (0.83 to 1.32)	0.72
Recurrence		<0.001
No recurrence	reference	
Non-CNS recurrence[c]	0.80 (0.65 to 0.99)	
CNS recurrence	2.57 (1.79 to 3.68)	
Metastatic Disease at Diagnosis (Yes)	0.73 (0.80 to 0.89)	0.002
Sex		<0.001
Male	Reference	
Female	1.31 (1.12 to 1.54)	
Age at Survey (years)		0.40
<40	Reference	
40-64	1.14 (0.94 to 1.40)	
≥65	1.09 (0.87 to 1.36)	
Time since Diagnosis (years)		0.18
<2	Reference	
2-5	1.00 (0.86 to 1.18)	
6-10	1.05 (0.83 to 1.32)	
≥10	0.74 (0.55 to 1.00)	
ESAS Tiredness (score ≥4)	1.74 (1.45 to 2.10)	<0.001
ESAS Depression (score ≥4)	1.89 (1.57 to 2.28)	<0.001
ESAS Pain (score ≥4)	1.18 (1.00 to 1.38)	0.04
ESAS Anxiety (score ≥4)	1.55 (1.29 to 1.87)	<0.001
ESAS Nausea (score ≥4)	0.88 (0.70 to 1.10)	0.25
ESAS Appetite (score ≥4)	1.08 (0.90 to 1.28)	0.41
ESAS Drowsiness (score ≥4)	1.64 (1.39 to 1.94)	<0.001
ESAS Shortness of Breath (score ≥4)	1.35 (1.14 to 1.60)	<0.001

Note: CNS, Central Nervous System; *ESAS*, Edmonton Symptom Assessment System (0-10, higher score indicates worse symptoms).
[a] high cognitive difficulty (quite a bit/very much)
[b] includes: Distant- other, Local – not CNS, Regional – not CNS, and unknown
[c] patients with primary brain cancers were not considered as having metastatic disease

© 2024 Mayo SJ et al. *JAMA Network Open*

eTable 5. Post-hoc Reduced Multivariable Analyses of Moderate to Severe Cognitive Symptoms by Cancer Type

A. Breast (n=816)

Effect	Moderate to Severe Cognitive Symptoms[a]	
	OR (95% CI)	p
**Chemotherapy**[b]		
Yes	1.53 (1.10 to 2.12)	0.01
No	Reference	
**ESAS Depression (score ≥4)**	1.99 (1.33 to 3.00)	<0.001
**ESAS Tiredness (score ≥4)**	1.66 (1.17 to 2.36)	0.005
**ESAS Anxiety (score ≥4)**	1.66 (1.10 to 2.50)	0.01
**ESAS Shortness of Breath (score ≥4)**	2.54 (1.67 to 3.85)	<0.001
**Metastatic Disease at Diagnosis (Yes)**	0.32 (0.13 to 0.78)	0.01

Note: 8 cases of total 824 cases in the *Breast Cancers* group not used due to missing data. Sex was not tested as a variable in this model, as all 816 cases had sex documented as female. *ESAS*, Edmonton Symptom Assessment System (0-10, higher score indicates worse symptoms).
[a] moderate to severe cognitive symptoms (quite a bit/very much), n=303
[b] refers to chemotherapy received as part of first-line treatment

B. Hematological Cancers (n=750)

Effect	Moderate to Severe Cognitive Symptoms[a]	
	OR (95% CI)	p
**Chemotherapy**[b]		
Yes	2.33 (1.35 to 4.02)	0.002
No	reference	
**Sex**		
Female	2.09 (1.45 to 3.00)	<0.001
Male	reference	
**ESAS Depression (score ≥4)**	1.81 (1.11 to 2.97)	0.02
**ESAS Tiredness (score ≥4)**	1.98 (1.19 to 3.28)	0.009
**ESAS Drowsiness (score ≥4)**	2.22 (1.42 to 3.47)	<0.001
**ESAS Anxiety (score ≥4)**	1.93 (1.19 to 3.13)	0.007

Note: 13 cases of total 763 cases in the *Hematological Cancers* group not used due to missing data. *ESAS*, Edmonton Symptom Assessment System (0-10, higher score indicates worse symptoms).
[a] moderate to severe cognitive symptoms (quite a bit/very much), n=213
[b] refers to chemotherapy received as part of first-line treatment

© 2024 Mayo SJ et al. *JAMA Network Open*

C. Head and Neck Cancers (n=580)

**Effect**	**Moderate to Severe Cognitive Symptoms[a]**	
	**OR (95% CI)**	**p**
**ESAS Depression (score ≥4)**	3.12 (2.02 to 4.83)	<0.001
**ESAS Tiredness (score ≥4)**	2.62 (1.63 to 4.22)	<0.001
**ESAS Shortness of Breath (score ≥4)**	1.81 (1.13 to 2.90)	0.01

Note: 5 cases of total 585 cases in the *Head and Neck Cancers* group not used due to missing data. *ESAS*, Edmonton Symptom Assessment System (0-10, higher score indicates worse symptoms).
[a] moderate to severe cognitive symptoms (quite a bit/very much), n=170

D. Gynecological Cancers (n=578)

**Effect**	**Moderate to Severe Cognitive Symptoms[a]**	
	**OR (95% CI)**	**p**
**Recurrence**		0.04
**No recurrence**	reference	
**Non-CNS recurrence[b]**		
**CNS recurrence**		
**ESAS Depression (score ≥4)**	4.04 (2.57 to 6.36)	<0.001
**ESAS Tiredness (score ≥4)**	2.23 (1.38 to 3.61)	0.001

Note: 5 cases of total 583 cases in the *Gynecological Cancers* group were not used due to missing data. Sex was not tested as a variable in this model, as all 578 cases had sex documented as female. *CNS*, Central Nervous System; *ESAS*, Edmonton Symptom Assessment System (0-10, higher score indicates worse symptoms).
[a] moderate to severe cognitive symptoms (quite a bit/very much), n=164
[b] includes Distant- other, Local – not CNS, Regional – not CNS, and unknown

E. Gastrointestinal Cancers (n=499)

**Effect**	**Moderate to Severe Cognitive Symptoms[a]**	
	**OR (95% CI)**	**p**
**ESAS Depression (score ≥4)**	2.28 (1.47 to 3.53)	<0.001
**ESAS Tiredness (score ≥4)**	2.63 (1.62 to 4.27)	<0.001

Note: ESAS, Edmonton Symptom Assessment System (0-10, higher score indicates worse symptoms).
[a] moderate to severe cognitive symptoms (quite a bit/very much), n=141
[b] refers to chemotherapy received as part of first-line treatment

F. Genitourinary Cancers (n=344)

**Effect**	**Moderate to Severe Cognitive Symptoms**[a]	
	**OR (95% CI)**	**p**
**ESAS Depression (score ≥4)**	2.02 (1.09 to 3.76)	0.03
**ESAS Tiredness (score ≥4)**	3.75 (1.96 to 7.17)	<0.001

Note: 4 cases of total 348 cases in the *Genitourinary Cancers* group not used due to missing data.
ESAS, Edmonton Symptom Assessment System (0-10, higher score indicates worse symptoms).
[a] moderate to severe cognitive symptoms (quite a bit/very much), n=73

G. Lung and Bronchus (n=331)

**Effect**	**Moderate to Severe Cognitive Symptoms**[a]	
	**OR (95% CI)**	**p**
**ESAS Drowsiness (score ≥4)**	2.15 (1.22 to 3.80)	0.009
**ESAS Anxiety (score ≥4)**	2.29 (1.30 to 4.02)	0.004

Note: 3 cases of total 334 cases in the *Lung and Bronchus* group not used due to missing data.
ESAS, Edmonton Symptom Assessment System (0-10, higher score indicates worse symptoms).
[a] moderate to severe cognitive symptoms (quite a bit/very much), n=81

H. Brain/CNS (n=313)

**Effect**	**Moderate to Severe Cognitive Symptoms**[a]	
	**OR (95% CI)**	**p**
**Age**		0.002
**<40**	reference	
**40-64**	3.27 (1.67 to 6.38)	
**≥65**	2.74 (1.14 to 6.60)	
**ESAS Depression (score ≥4)**	2.34 (1.35 to 4.06)	0.003
**ESAS Tiredness (score ≥4)**	3.03 (1.69 to 5.44)	<0.001

Note: 1 case of total 314 cases in the *Brain/CNS* group were not used due to missing data. Recurrence was not tested as a variable in this model, as all 313 patients had documented CNS recurrence. *CNS*, Central Nervous System; *ESAS*, Edmonton Symptom Assessment System (0-10, higher score indicates worse symptoms).
[a] moderate to severe cognitive symptoms (quite a bit/very much), n=160

© 2024 Mayo SJ et al. *JAMA Network Open*

I. Sarcoma (n=202)

Effect	Moderate to Severe Cognitive Symptoms[a]	
	OR (95% CI)	p
**ESAS Depression (score ≥4)**	3.61 (1.60 to 8.14)	0.002
**ESAS Tiredness (score ≥4)**	2.88 (1.19 to 6.95)	0.02

Note: 3 cases of total 205 cases in the *Sarcoma* group not used due to missing data. *ESAS*, Edmonton Symptom Assessment System (0-10, higher score indicates worse symptoms).
[a] moderate to severe cognitive symptoms(quite a bit/very much), n=50

J. Thyroid (n=185)

Effect	Moderate to Severe Cognitive Symptoms[a]	
	OR (95% CI)	p
**ESAS Depression (score ≥4)**	2.81 (1.35 to 5.86)	0.006
**ESAS Tiredness (score ≥4)**	5.36 (1.67 to 17.28)	0.005
**ESAS Drowsiness (score ≥4)**	2.43 (1.04 to 5.68)	0.04

Note: ESAS, Edmonton Symptom Assessment System (0-10, higher score indicates worse symptoms).
[a] moderate to severe cognitive symptoms (quite a bit/very much), n=67

K. Melanoma (n=94)

Effect	Moderate to Severe Cognitive Symptoms[a]	
	OR (95% CI)	p
**ESAS Depression (score ≥4)**	3.42 (1.19 to 9.82)	0.02
**ESAS Tiredness (score ≥4)**	2.45 (0.83 to 7.20)	0.10

Note: ESAS, Edmonton Symptom Assessment System (0-10, higher score indicates worse symptoms).
[a] moderate to severe cognitive symptoms (quite a bit/very much), n=29

L. All other Cancers (n=385)

Effect	Moderate to Severe Cognitive Symptoms[a]	
	OR (95% CI)	p
**Sex**		
Female	1.86 (1.13 to 3.07)	0.01
Male	reference	
**ESAS Depression (score ≥4)**	2.71 (1.60 to 4.60)	<0.001
**ESAS Tiredness (score ≥4)**	1.94 (1.06 to 3.56)	0.03
**ESAS Shortness of Breath (score ≥4)**	2.06 (1.18 to 3.62)	0.015

Note: 4 cases of total 385 cases in the *All Other Cancers* group not used due to missing data. *ESAS*, Edmonton Symptom Assessment System (0-10, higher score indicates worse symptoms).
[a] moderate to severe cognitive symptoms (quite a bit/very much), n=102

© 2024 Mayo SJ et al. *JAMA Network Open*
