# Supplementary material for: Cognitive Symptoms Across Diverse Cancers
Source: JAMA Netw Open. 2024 Aug 28;7(8):e2430833. doi: 10.1001/jamanetworkopen.2024.30833 (PMC11358862; doi:10.1001/jamanetworkopen.2024.30833)
Supplement: Supplement 2. — Data Sharing Statement [file jamanetwopen-e2430833-s002.pdf]

Data Sharing Statement

Mayo. Cognitive Symptoms Across Diverse Cancers. *JAMA Netw Open*. Published August 28, 2024. doi:10.1001/jamanetworkopen.2024.30833

Data
Data available: No

Additional Information
Explanation for why data not available: The data underlying this article are not publicly available due to privacy and ethical restrictions.
